# Clinical Outlook for Type-1 and FOXP3^+^ T Regulatory Cell-Based Therapy

**DOI:** 10.3389/fimmu.2015.00593

**Published:** 2015-11-25

**Authors:** Silvia Gregori, Laura Passerini, Maria-Grazia Roncarolo

**Affiliations:** ^1^Division of Regenerative Medicine, Stem Cells and Gene Therapy, IRCCS San Raffaele Scientific Institute, San Raffaele Telethon Institute for Gene Therapy (TIGET), Milan, Italy; ^2^Department of Pediatric Stem Cell Transplantation and Regenerative Medicine, Stanford School of Medicine, Palo Alto, CA, USA

**Keywords:** T regulatory cells, T regulatory type 1 cells, tolerance, Treg-based therapy, IL-10, FOXP3, gene transfer

## Abstract

T regulatory cells (Tregs) are subsets of T lymphocytes specialized in modulating antigen-specific immune responses *in vivo*. Hence, Tregs represent an ideal therapeutic tool to control detrimental immune reactions. Based on solid pre-clinical results, investigators started testing the safety and efficacy of Treg-based therapies in humans. Despite promising results, a number of issues remain to be solved. We will discuss the results obtained from clinical trials and the challenges and risks we are facing in the further development of Treg-based therapies.

## Introduction

**Figure 1:**
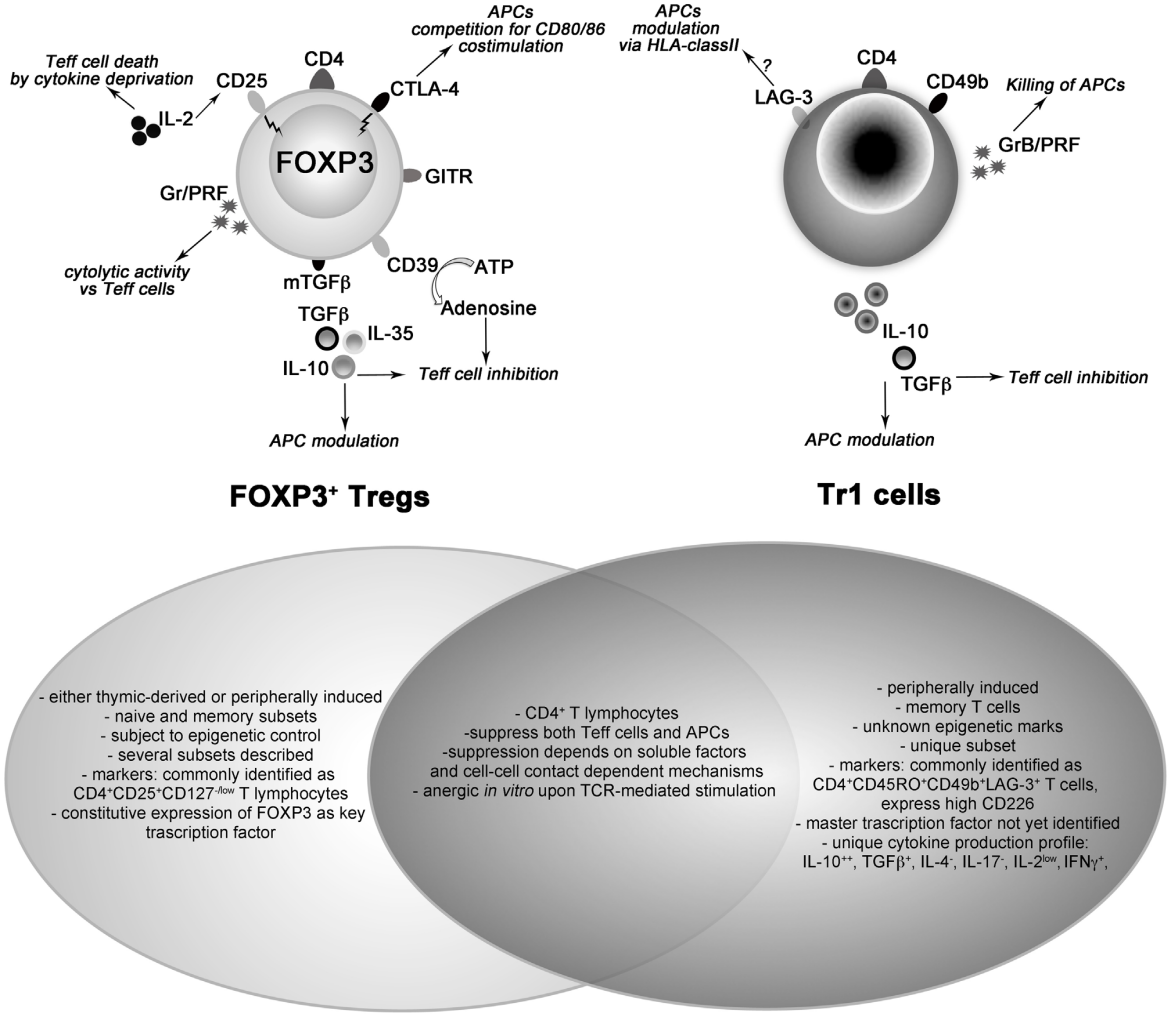
**Schematic representation of cell surface and intracytoplasmic markers and mechanisms of action characterizing FOXP3-expressing (left cartoon) and type 1 (right cartoon) T regulatory cells**. The shared and unique features of both cell types are listed in the frames. Ag, antigen; APC, antigen-presenting cell; ATP, adenosine triphosphate; CTLA-4, cytotoxic T-lymphocyte antigen 4; FOXP3, forkhead box protein 3; GITR, glucocorticoid-induced TNFR family related gene; Gr; Granzymes; LAG-3, lymphocyte-activation gene 3; PRF, perforin; Teff, effector T cell; TGFβ, transforming growth factor β; Tr1, type 1 T regulatory cell.

T regulatory cells (Tregs) are a component of the immune system involved in modulating immune reactions and in inducing tolerance. Due to their potential as immune modulators, therapeutic application of Tregs to control undesirable immune responses and to promote tolerance has become an active field of investigation (1). Over the years, several types of Tregs have been identified, and the forkhead box P3 (FOXP3)-expressing Tregs (FOXP3^+^ Tregs) (2) and the T regulatory type 1 (Tr1) cells (3) are the best characterized (Figure 1).

FOXP3^+^ Tregs can be either thymus-derived (tTregs), or induced in the periphery (pTregs) (4, 5). Regardless of their origin, both subsets are characterized by constitutive expression of the IL-2Rα-chain (CD25), in the absence of the IL-7Rα-chain (CD127), and of FOXP3 (6), making the two subsets indistinguishable based on their phenotype. High expression of Helios has been identified in FOXP3^+^ Tregs (7), and suggested to be specific for tTregs (8). However, this notion was later challenged by the demonstration that Helios is also expressed by non-tTregs (9, 10). To date, the most reliable feature unambiguously identifying tTregs is the epigenetic remodeling of a specific region in the *FOXP3* locus, indicated as Treg-specific-demethylated-region (TSDR) (11). A more comprehensive CpG hypomethylation pattern of tTregs including several Treg-related genes has been described (12).

In addition to CD25, along the years, the expression of several molecules, i.e., CTLA-4 (13), GITR (14), CD39 (15), Galectin 10 (16), latency-associated-peptide (LAP) (17), and glycoproteinA-repetitions-predominant (GARP) (18) has been attributed to human FOXP3^+^ Tregs. The expression of the above-mentioned molecules is not exclusive to FOXP3^+^ Tregs, since they are often shared with activated conventional T cells.

CTLA-4, GITR, and CD39 are specifically associated with FOXP3^+^ Treg suppressive function, which is primarily dependent on contact with target cells. Additional mechanisms of suppression have been described for FOXP3^+^ Tregs, including release of IL-10 (19), TGF-β (20, 21), and IL-35 (22), direct killing of T effector (Teff) cells through the granzyme/perforin axis (23), modulation of antigen-presenting cells (APCs) stimulatory capacity *via* CTLA-4 (24), cytokine deprivation (25), and generation of immunosuppressive metabolites, such as extracellular adenosine (26) and intracellular cAMP (27). The variety of phenotypes and weapons discovered led from the original idea of FOXP3^+^ Tregs as homogeneous population to the modern view of a heterogeneous pool, including several specialized subtypes characterized by expression of specific cell surface markers such as ICOS (19), HLA-DR (28, 29), and CD45 isoforms (30, 31).

Tr1 cells are memory T lymphocytes expressing CD49b and LAG-3 (32). Tr1 cells, upon activation, secrete high levels of IL-10 and TGF-β, variable amounts of IL-5, GM-CSF, and IFN-γ, and minimal amounts of IL-2, IL-4, and IL-17 (3, 33, 34). Tr1 cells express CTLA-4, (35, 36), PD-1 (36), and ICOS (37). Similar to FOXP3^+^ Tregs, Tr1 cells can express CD39 and CD73 [Ref. (38–41) and (Gregori et al. unpublished data)]. Tr1 cells do not constitutively express FOXP3 (42), thus they are distinct from both tTregs and pTregs; however, upon activation, Tr1 cells can transiently up-regulate FOXP3, but its expression never reaches the levels of FOXP3^+^ Tregs (33, 43–45).

The main mechanism by which Tr1 cells control immune responses is the secretion of IL-10 and TGF-β. Importantly, to exert their suppressive function, Tr1 cells need to be activated *via* their TCR, but, once activated, they can mediate bystander suppressive activity against other antigen(Ag)s (3, 33). IL-10 and TGF-β directly inhibit T-cell responses by suppressing IL-2 and IFN-γ production and T-cell proliferation, and indirectly act on APCs by down-modulating costimulatory molecules, HLA-class-II, and pro-inflammatory cytokine production (34). In addition to the cytokine-mediated suppression, Tr1 cells inhibit T-cell responses by killing myeloid APCs *via* granzyme B (46). Tr1 cell-mediated cytotoxicity of myeloid APCs requires stable adhesion with target cells and activation *via* HLA-class-I molecules and CD112/CD155 expressed on target cells (46). New evidence suggests that Tr1 cells use additional modes of immune regulation to achieve tolerance: they can inhibit T-cell responses by cell-contact dependent mechanisms (36) and by metabolic disruption (33, 39, 41).

Results from pre-clinical murine and humanized models convinced investigators that Tregs can be used to control graft-versus-host disease (GvHD) as well as organ rejection, or to treat autoimmune diseases (47, 48). Good-manufacturing-practice (GMP)-grade protocols to isolate and expand human Tregs *in vitro* without losing their suppressive function and to generate human Ag-specific Tregs have been established allowing translation of Treg-based therapy to the clinical practice.

## Completed and Ongoing Treg-Based Clinical Trials

Treg-based therapy has been used for the first time to prevent GvHD in patients undergoing allogeneic hematopoietic stem cell transplantation (allo-HSCT). Six independent trials, using either FOXP3^+^ Tregs or Tr1 cells, have been concluded, and all of them showed the feasibility and safety of Treg-based approaches (49–54) (Table 1). In five of these trials, either freshly isolated (51, 54, 55) or *ex vivo* expanded FOXP3^+^ Tregs (49, 50) were infused in patients undergoing allo-HSCT for onco-hematological diseases. Three of these trials also indicated the potential efficacy of the treatment. Brunstein et al. (50) reported a decreased incidence of grade II–IV GvHD as compared to historical controls when umbilical cord blood (UBC)-derived Tregs were injected, without increased risk of infections. Similarly, Di Ianni et al. (51) described few cases of low grade GvHD (2 out of 26 patients) and no development of chronic GvHD in patients injected with un-manipulated peripheral Tregs. More recently, it has been reported that in Treg-treated patients, the cumulative incidence of relapse was significantly lower than in historical controls (54). Previous trials based on the adoptive transfer of alloAgs-specific anergic T cells generated *in vitro* in the presence of Belatacept (CTLA-4-Ig) to prevent GvHD after allo-HSCT were performed (56, 57). Later, it was demonstrated that alloAgs-specific anergic T cells generated with CTLA-4-Ig contained a small fraction of FOXP3^+^ Tregs (58).

**Table 1:** **Completed Treg-based clinical trials**.

Trial ID	Cell product	Disease	Safety	Efficacy	Reference
N.A.	*In vitro* expanded donor-derived CD4^+^CD25^high^CD127^−^ Tregs	GvHD after HLA-matched sibling HSCT for hematological malignancies	Yes	N.A.	(49)
N.A.	Freshly isolated donor-derived CD4^+^CD25^high^CD127^−^ Tregs	GvHD after allo-HSCT for hematological malignancies	Yes	N.A.	(55)
NCT00602693	*In vitro* expanded UCB-derived CD4^+^CD25^+^ Tregs	GvHD after DUCBT for hematological malignancies	Yes	Yes	(50)
CEAS Umbria Protocol No 01/08 2008	Freshly isolated donor-derived CD4^+^CD25^+^ Tregs	GvHD after haplo-HSCT for hematological malignancies	Yes	Yes	(51)
CEAS Umbria Protocol No 0108	Freshly isolated donor-derived CD4^+^CD25^+^ Tregs	GvHD after haplo-HSCT for hematological malignancies	Yes	Yes	(54)
NKEBN/8/2010	*In vitro* expanded autologous CD4^+^CD25^high^CD127^−^ Tregs	Pediatric recent onset T1D	Yes	N.A.	(59)
ALT-TEN	IL-10 DLI donor-derived IL-10 anergized T cells	GvHD after haplo-HSCT for hematological malignancies	yes	N.A.	(53)
CATS 1	Autologous OVA-specific Tr1 cell clones; Ovasave^®^	Refractory Crohn’s Disease	Yes	Yes	(40)

*GvHD, graft-versus-host disease; haplo-HSCT, haploidentical-hematopoietic stem cell transplantation; UCB, umbilical cord blood; DUCBT, double umbilical cord blood transplant; allo-HSCT, allogeneic-HSCT; T1D, type 1 diabetes; DLI, donor lymphocyte infusion; OVA, ovalbumin*.

Our group has completed a phase-I clinical trial in which IL-10-anergized T cells (IL-10 DLI) containing Tr1 cells were injected in patients undergoing haploidentical-HSCT (53). Donor-derived IL-10-anergized T cells specific for host allo-Ags were generated *in vitro* through activation of T cells by host-derived APCs in the presence of exogenous IL-10 (60). An improved protocol for the generation of Tr1 cells, which foresees the use of tolerogenic dendritic cells (DC-10)(61), has been developed (60, 62). Although a small cohort of patients was treated, our results demonstrated that after infusion of IL-10 DLI no acute adverse events and only mild GvHD (grade II or III responsive to therapy) were observed. Furthermore, the treatment accelerated immune reconstitution after transplant and long-lasting disease remission (53).

The above-mentioned trials paved the way to a wider application of Tregs as advanced medical products for the treatment of autoimmunity in type 1 diabetes (T1D), inflammatory diseases, and rejection after solid organ transplantation. *Ex vivo* expanded CD4^+^CD25^hi^CD127^−^ Tregs were administered to children with recent onset T1D in a phase-I trial (59) (Table 1). The procedure appeared to be safe, as no adverse reactions related to the treatment were reported. However, the few data available do not allow drawing conclusions on the clinical relevance of the procedure (59). The group of Bluestone is currently testing the safety of *ex vivo* expanded polyclonal CD4^+^CD25^hi^CD127^low/−^ Tregs in a phase-I clinical trial (NCT01210664) in which increasing doses of Tregs will be injected in recent onset adult T1D patients (63). A phase-I/IIa clinical study in which Ag-specific Tr1 cell clones were used to treat patients with Crohn’s Disease has been recently reported. Overall, a response was observed in 40% of patients, with stronger effect in the group of patients who received the lowest Tr1 cell dose (40) (Table 1). The France-based company TxCell is currently heading a consortium dedicated to the clinical development of collagen-specific Tr1 cells (Col-Treg) to be tested in a first-in-man clinical study for severe and refractory autoimmune uveitis scheduled to start in 2016.^1^

The power of Tregs in inducing tolerance to allo-Ags after solid organ transplantation is currently under evaluation. In liver transplantation, several clinical trials are ongoing using polyclonal expanded Tregs with or without rapamycin (Treg trial, NCT01624077, ThRIL trial NCT02166177) or donor-specific expanded Tregs (darTreg: deLTA Trial NCT02188719, and ARTEMIS Trial NCT02474199). In addition, *ex vivo* expanded autologous polyclonal CD4^+^CD25^+^ Tregs are currently tested in the context of kidney transplantation (TRACT Trial, NCT 02145325 and TASK Trial NCT0288931). Moreover, an ambitious project in which the efficacy of different immune-regulatory cells, including polyclonal expanded Tregs with or without rapamycin (One Treg1 Trial, NCT02129881, ONE nTreg13 Trial NCT02371434), darTreg cells (DART Trial NCT02244801), and donor-specific T cells anergized in the presence of Belatacept (NCT02091232), and Tr1 cells induced with DC-10, will be compared in kidney transplant ­recipients (“The ONE study,” discussed in details below) is currently ongoing. Results of these trials will definitely address the safety of this approach and will also provide hints on their efficacy as therapeutic agents.

## Open Issues in Treg-Based Immunotherapy

Despite the promising results obtained from the above-mentioned pilot clinical trials, many open questions remain on the best source and subtype of Tregs to be administered, the survival of these cells in the host, and their mechanisms of action.

The ONE Study^2^ is a large-scale, collaborative project funded by the Seventh Framework Programme (FP7) of the European Commission, envisioned to ascertain which immuno-modulatory cell type (among *ex vivo* isolated and *in vitro* expanded polyclonal or allo-specific FOXP3^+^ Tregs, Tr1 cells, and tolerogenic APCs) is best fit to induce tolerance to allo-Ags in patients receiving kidney transplants (64, 65). Results from this study will define which regulatory cell population is the most efficient in promoting graft acceptance and tolerance.

Recent work has led to the identification of specialized subsets of Tregs, which reside in peripheral tissues, including skin, intestinal mucosa, adipose tissue, autoimmune target tissues, and injured muscle (66). Although tissue-resident Tregs represent a small fraction of total Tregs, their peculiar phenotype and function confer the ability to regulate tissue-specific physiological and pathological processes. Therapies aimed at targeting tissue-specific Tregs may potentially allow the local control of the disease, without affecting systemic immunity. Although the clinical application of tissue-resident Tregs remains unexplored, the possibility of exploiting these subsets deserves to be investigated in the near future.

One pre-requisite for Treg-based therapies is their *in vivo* viability and persistence. In a clinical trial in allo-HSCT, upon *in vivo* infusion Tregs were no longer detected in the circulation after 2 weeks (50). Similarly, in T1D patients, *in vitro* expanded CD4^+^CD25^+^CD127^−^ Tregs labeled with deuterium were found at high frequency in the peripheral blood 2 weeks after injection, then declined but they were still detectable at low frequency 6 months after therapy [Bluestone JA, unpublished data presented at FOCIS Annual Meeting 2015]. It is still unclear whether infused Tregs migrate to tissues or have limited *in vivo* survival because of *in vitro* expansion. In IL-10 DLI-treated patients, we found an expansion of circulating granzyme B/IL-10 and CD49b/LAG-3-expressing CD4^+^ T cells that progressively increased during follow up. The percentages of these cells were higher in the IL-10 DLI-treated long-term surviving patients (up to 8 years after haplo-HSCT), as compared to those in healthy subjects (53). These data support the hypothesis that IL-10 DLI infusion supports either Tr1 cell expansion, or the *de novo* induction of Tr1 cells.

Increasing evidence suggests that FOXP3-expressing Tregs are intrinsically plastic (67–69). Therefore, the risk of their *in vivo* conversion into Teff cells under inflammatory conditions, and consequent loss of their suppressive ability, cannot be ignored. To allow safe clinical application of Tregs, investigators are currently trying to address this issue. For example, rapamycin permits the *in vitro* expansion of FOXP3^+^ Tregs, while impairing the proliferation of contaminating Teff cells (70, 71). Importantly, rapamycin-expanded FOXP3^+^ Tregs maintain their regulatory phenotype, even upon exposure to a pro-inflammatory environment (72, 73). Clinical-grade Treg expansion protocols with rapamycin have been implemented for ongoing clinical trials under the umbrella of the European consortium “The ONE study” (65, 74). On the same line, in order to avoid infusion of Teff cell contaminants potentially allo-reactive, allo-anergization of T cells in the presence of costimulatory blockade with Belatacept has been proposed (58) and is currently being tested (NCT02091232).

One major concern for the use of immunotherapy with Tregs to control GvHD after allo-HSCT for hematological malignancies is the potential inhibition of the beneficial graft-versus-leukemia (GvL) effects. Results from one of the completed phase II trials showed that in CD25^+^ Treg-treated patients the cumulative incidence of relapse was significantly lower than in historical controls. The Authors proposed that the failure of human CD4^+^CD25^+^ Tregs to home to the bone marrow does not hamper the GvL activity of the donor conventional T cells (54). Although promising, these results are still preliminary and required further confirmation.

## Up-Coming Challenges in Treg-Based Immunotherapy

As previously mentioned, increasing evidence suggests that FOXP3^+^ Tregs are a heterogeneous population, including several specialized subtypes, making it difficult to choose the “right” variety of cells for specific treatments. To overcome this limitation, we developed a novel and efficient method to generate homogeneous populations of human FOXP3-expressing Tregs by Lentiviral-Vector (LV)-mediated *hFOXP3* gene transfer into conventional CD4^+^ T cells, hereafter indicated as CD4^FOXP3^ T cells. Constitutive over-expression of FOXP3 generates functional and stable FOXP3^+^ Treg-like cells, with potent *in vitro* and *in vivo* suppressive activity, reduced proliferative capacity and cytokine production (75, 76). CD4^FOXP3^ T cells generated from naïve CD4^+^ T cells have stable expression of FOXP3 in steady state and inflammatory conditions, whereas CD4^FOXP3^ T cells generated from memory cells show reduced percentage of FOXP3^+^ T cells upon activation, especially in the presence of inflammatory cytokines. The instability of FOXP3 expression in memory CD4^FOXP3^ T cells results in weaker suppressive function and increased proliferative capacity, confirming that acquisition of Treg functions is dependent on stable FOXP3 expression (76).

Despite recent advances in the establishment of protocols to efficiently generate Allo Ag-specific Tr1 cells *in vitro*, the resulting populations still contain contaminants that could potentially limit the *in vivo* efficacy of Tr1 cells (60, 61). The recent discovery of CD49b and LAG-3 as specific biomarkers of Tr1 cells that allow the isolation of Tr1 cells from *in vitro* Tr1-polarized populations (32) will open the possibility to select human Tr1 cells from mixed cultures. As an alternative to obtain a large and homogeneous population of Tr1 cells, the LV-mediated *hIL-10* gene transfer has been used to convert conventional T cells into Tr1-like cells, termed CD4^IL-10^ (77). CD4^IL-10^ cells mirror the phenotype and function of Tr1 cells and suppress xeno-GvHD (77). These findings pave the way for adoptive cell therapy with FOXP3- or IL-10-engineered T cells in patients with autoimmune disorders and in patients undergoing allogeneic organ or HSC transplantation. Issues related to undesired effects of therapy with genetically modified cells, such as induction of general immunosuppression, impairment of immune reconstitution, and GvL activity in the context of allo-HSCT for hematological diseases are still under investigation.

In humanized pre-clinical models, allo-specific Tregs are more effective in preventing graft rejection as compared to polyclonal Tregs (78, 79). It is possible to select allo-specific Tregs from peripheral blood according to the expression of early activation markers and/or then *in vitro* expand them (78, 80). Moreover, a GMP-grade protocol to selectively expand human allo-specific Tregs using CD40L-activated B cells has also been established (79). As an alternative, ectopic expression of genes encoding for TCR with known specificity has been proposed. Forced expression of specific TCRs confers the desired specificity to human polyclonal Tregs. As a proof-of-concept, it has been shown that TCR or chimeric receptor specific for tumor Ags can be introduced in human polyclonal Tregs, conferring them the ability to potently suppress anti-tumor responses (81–83). It was also proposed to generate Ag-specific Tregs starting from conventional T cells engineered to over-express both *hFOXP3* and TCR specific for a birch pollen allergen-derived peptide Betv1. The resulting T cells acquired a Treg phenotype and suppressed T-cell responses in an Ag-specific manner (84). Despite these data provided the proof-of-concept for such approaches, several questions regarding the potential clinical application of these engeneered T cells have to be addressed. Among others, one of the major concerns regards the need to eliminate endogenous TCRs to avoid double specificity and the risk of bystander undesired suppressive function. An interesting and promising approach to overcome this limitation is LV-mediated gene transfer of either *hFOXP3* or *hIL-10* in Ag-experienced T cells isolated from peripheral blood.

An additional crucial question for the success of Treg-based therapy, in particular in the context of solid organ transplantation, is how immunosuppressive treatments affect Treg survival and function. The impact of current immunosuppressive drugs on Tregs has been extensively reviewed in Ref. (48, 85). The general consensus is that calcineurin inhibitors are likely to be detrimental to Tregs, whereas drugs such as rapamycin or mycophenolate mofetil (MMF) preserve Tregs *in vivo*. However, indications will come from the results of “The ONE study” in which Tregs will be infused in patients receiving kidney transplantation and standard triple-therapy protocol (prednisolone, MMF, and tacrolimus) (65).

Finally, the heterogeneity of the parameters selected to monitor Treg activity in the recently completed trials hampers comparison of the results. To overcome this limitation, the EU COST Action “BM1305: action to focus and accelerate cell-based tolerance-inducing therapies^3^” has been funded to identify shared and disease specific biomarkers of tolerance in patients undergoing Treg-based therapies. This action is complementary to “The ONE study” and aims at defining general tolerance signatures and standardized immune monitoring protocols (65).

## Concluding Remarks

The discovery that Tregs modulate immune responses led to the idea that they could be developed as a therapeutic tool to promote/restore tolerance to transplanted grafts and in inflammatory and autoimmune diseases. The recent clinical trials proved the safety of this approach and suggested a possible therapeutic effect. Thus far, the major challenges in the field were to expand hard-to-grow polyclonal Tregs to great purity, and to generate Ag-specific Tregs. Despite technical advances in the field, many questions relating to Treg-based therapies remain unanswered: Which cell type to be used? Which schedule of cell infusion? How long Tregs will survive *in vivo*? How long their effect will last? What is their mechanism of action? Do they interfere with GvL in the context of allo-HSCT? Moreover, reliable biomarkers of tolerance and standardized methods to evaluate the efficacy of Treg-based therapy are required to compare the outcome of present and future trials. To address these questions, close collaboration between groups in the field is required to allow the systematic comparison of Tregs and outcomes of cell therapy trials.

## Conflict of Interest Statement

The authors declare that the research was conducted in the absence of any commercial or financial relationships that could be construed as a potential conflict of interest.

## Footnotes

^1^http://www.txcell.com

^2^www.onestudy.org

^3^www.afactt.eu
